# Supplementary material for: Novel Viruses in Mosquitoes from Brazilian Pantanal
Source: Viruses. 2019 Oct 17;11(10):957. doi: 10.3390/v11100957 (PMC6832572; doi:10.3390/v11100957)
Supplement: Supplementary file 1 [file viruses-11-00957-s001.zip › Suplementary table 1.docx]

Suplementary table 1: Oligonucleotides designed to amplify putative novel viral sequences identified in salivary glands of mosquitoes from Pantanal, Brazil.

| **Target** | **Genomic region** | **Primer sequence** | **Estimated size (bp)** | **Genome position** |
| --- | --- | --- | --- | --- |
| Iflavirus-like | C-terminal | Ifla1F: AACACGTTCCTCCTGTTGGG | 300 | 7,285-7418 |
|  |  | Ifla1R: TGTTTAGACTTAGGCGGCGG |  |  |
| Iflavirus-like | C-terminal | Ifla2F: TGCGGAACAAATTCATTGCGAT | 1500 | 5,400-6,918 |
|  |  | Ifla2R: ACAACGAACGGAAACCATCTCT |  |  |
| Rhabdovirus-like | M-G* | MGMF: TGGCTTGAAAACATCTATTCAGGC | 1000 | 1,319-1,980 |
|  |  | MGMR: TGCTACTACTGCAGTGAATCCG |  |  |
| Rhabdovirus-like | G-L | GLMF: ACAGCTTACGACCGACATC | 1000 | 1,900-2,810 |
|  |  | GLMR: GAAACTGCCTGATGGAGGGG |  |  |
| Circovirus-like | Rep-Cap** | CircoF: AAATTTAATCGAGAAACCAGTAACTTTGAA | 1000 | 100-500 |
|  |  | CircoR: AATCTTGTTTTTCCGACTCCGCTT |  |  |

*Region between M-G, G-L genes. **Forward primer designed to Rep: Replication associated protein; reverse primer designed to Cap: Capsid protein (genome with circular topology).
